# Accumulation and translocation of lead in vegetables through intensive use of organic manure and mineral fertilizers with wastewater

**DOI:** 10.1038/s41598-024-63076-x

**Published:** 2024-06-02

**Authors:** Mehwish Amjad, Zafar Iqbal Khan, Muhammad Nadeem, Kafeel Ahmad, Anis Ali Shah, Mansour K. Gatasheh, Shifa Shaffique, Toqeer Abbas

**Affiliations:** 1 Department of Botany, University of Sargodha, Sargodha, Pakistan; 2 Institute of Food Science and Nutrition, University of Sargodha, Sargodha, Pakistan; 3 Department of Botany, Division of Science and Technology, University of Education, Lahore, Punjab Pakistan; 4 Department of Biochemistry, College of Science, King Saud University, P.O.Box 2455, 11451 Riyadh, Saudi Arabia; 5 College of Agriculture and Life Science, School of Applied Biosciences, Kyungpook National University, 80 Daehak-ro, Buk-gu, Daegu, 41566 Korea

**Keywords:** Pollution indices, Enrichment factor, Wastewater, Organic and inorganic fertilizers, Sargodha, Ecology, Physiology, Plant sciences, Environmental sciences

## Abstract

In many countries with wastewater irrigation and intensive use of fertilizers (minerals and organics), heavy metal deposition by crops is regarded as a major environmental concern. A study was conducted to determine the impact of mineral fertilizers, cow manure, poultry manure, leaf litter, and sugarcane bagasse on soil’s trace Pb content and edible parts of vegetables. It also evaluated the risk of lead (Pb) contamination in water, soil, and food crops. Six vegetables (*Daucus carota, Brassica oleracea, Pisum sativum, Solanum tuberosum, Raphanus sativus,* and *Spinacia oleracea*) were grown in the field under twelve treatments with different nutrient and water inputs. The lead concentrations in soil, vegetables for all treatments and water samples ranged from 1.038–10.478, 0.09346–9.0639 mg/kg and 0.036–0.26448 mg/L, The concentration of lead in soil treated with wastewater in treatment (T6) and vegetable samples was significantly higher, exceeding the WHO’s permitted limit. Mineral and organic fertilizers combined with wastewater treatment reduced lead (Pb) concentrations in vegetables compared to wastewater application without organic fertilizers. Health risk indexes for all treatments except wastewater treatment (T_6_) were less than one. Pb concentrations in mineral fertilizers, cow manure, poultry manure, leaf litter, and sugarcane bagasse treated were determined to pose no possible risk to consumers.

## Introduction

Wastewater could potentially offer a more consistent and nutrient-rich source for irrigation compared to both rainfall and groundwater^1^. Farmers choose to use wastewater for irrigation instead of freshwater because it leads to better crop yields, even though freshwater is available in some places^2^. It’s important to mention that the levels of heavy metals in wastewater discharges are higher than those found in groundwater^3^. Irrigation water, inorganic fertilizer, and compost-based organic fertilization are other pollutant sources^4^. The productivity of agricultural crops diminishes as heavy metals hinder the efficient transfer of nutrients^5^.

Lead (Pb) is a persistent and toxic substance commonly found in water due to its leaching from durable materials. However, significant amounts of Pb can also originate from battery smelters and household piping^6^. While Pb does occur naturally in the Earth’s crust, the majority of Pb concentrations in the environment are attributed to human activities^7^. The introduction of tetraethyl lead (TEL) as an anti-knocking agent in gasoline has led to the emergence of an artificial lead cycle^8^. In automobile engines, lead is consumed to create lead salts such as chlorides, bromides, and oxides. These lead salts are then emitted into the environment through vehicle exhaust^9^. While heavier particles settle quickly, contaminating soils or surface waterways, smaller particles disperse over greater distances in the air and remain in the environment for extended periods^10^. Rainfall causes some of this lead to return to the ground. This human-induced lead cycle is considerably longer than the natural environmental lead cycle^11^.

Lead tightly binds to soil particles, accumulating primarily in the top two inches when undisturbed. Soil serves as a major accumulator of lead from various sources, including fine particles that can be ingested as soil dust^12^. Lead in soil can react with other components to form lead-containing minerals. Wastewater and airborne contaminants are potential contributors to elevated soil lead levels. Implementing phytoremediation plants and reducing wastewater irrigation can help maintain acceptable soil lead levels, as polluted water used for irrigation is a significant source of lead uptake by plants^13^.

Potential of Stigmatocarpum criniflorum and Pelargonium hortorum for lead (Pb) phytoremediation. Both plants were exposed to Pb-contaminated soils ranging from 500 to 2000 mg/kg for duration of three weeks^14^. The research found that *P. hortorum* exhibited a two-fold greater accessible Pb fraction in the rhizosphere (for 2000 mg/kg Pb), indicating its superior ability to remediate Pb-polluted soil compared to *S. criniflorum*. *P. hortorum* showed higher Pb accumulation per plant when compared to *S. criniflorum*. Consequently, the authors suggested the utilization of *P. hortorum* for the treatment of Pb-polluted soils.

Plants have a limited capacity to effectively accumulate lead from the soil, although they can occasionally do so in high proportions^15^. Lead tends not to accumulate rapidly in areas where vegetables and food crops are grown. In biological processes, lead often displaces other metals like zinc, calcium, and iron, significantly increasing its potential harm. This displacement can lead to interference with proteins influencing genetic makeup and instability of the nervous system. Lead contamination poses a significant threat to approximately 26 m lives annually, resulting in 540,000 deaths, with the majority doccurring in poorer countries^16^. Lead (Pb) was found in toxic concentrations in four ecotypes when checking heavy metals in industrial effluents^17^.

Consequently, monitoring the toxicity levels of lead (Pb) metal is essential, as it determines the potential hazards posed by Pb to both plants and human health in the environment. The authors aimed to investigate lead hazards in food crop consumers and the transmission of lead along the water-soil-plant continuum under different treatments using various irrigation water sources.

This study was carried out to investigate how harmful lead (Pb) concentration is in water, soil, and vegetables, (ii) to assess the level of contamination through the intensive use of organic and inorganic fertilizers, (iii) to determine various mobility and pollution indices, including health risk, that is very important for restoring and securely preserving the public from polluted vegetables, and (iv) application of mineral fertilizers can decrease the Pb toxicity level in different vegetables.

## Materials and methods

### Experimental site

The experimental work was carried out in Chak # 89 S.B, situated in District Sargodha, central Punjab, Pakistan. The geographical coordinates for this study were 32.083° N and 72.6719° E. An average of 410 mm of rain falls in the area each year. It is situated at a height of around 190 m above sea level, with a mean temperature of 23.8 °C being recorded.

### Treatment details and experimental design

#### Treatments

The study comprised 12 treatment combinations of four levels of organic manure (control, cow manure at a rate of 20 t ha^−1^, poultry manure at a rate of 3 t ha^−1^, leaf litter at the rate of 50 t ha^−1^, sugarcane bagasse ash at a rate of 40 t ha^−1^) and one inorganic fertilizers (nitrate) at a rate of 100 kg ha^−1^) with irrigation of canal water and wastewater.

#### Field preparation

The field of study was ploughed fully and transversely furrowed using a mold sheet plow, and cross-scouring was performed with a tractor. The softened ground was set out in an experimental layout. Next, planking was completed, and the soil was brought to a fine tilth. Paths and canals were also prepared in 2.4 × 1.8 m^2^ plots according to the design. Cow manure, Poultry manure, leaf litter manure, and sugarcane bagasse manure were utilized at a rate of 20 t ha^−1^, 3 t ha^−1^, 50 t ha^−1^, and 40 t ha^−1^, and spread uniformly in a bed size of 2.4 × 1.8 m^2^. Its quantity was calculated and applied before sowing. The recommended dose of nitrate for vegetables was 100 kg ha^−1^. Experiments were carried out on selected vegetables for two yrs. A completely randomized design was used for the trial, with 12 treatments, five replicates, and six vegetables (12 × 5 × 6) (Table 1). The research consisted of field experiments in the work location and under the same environmental conditions. At maturity, fruit parts of vegetables were used for Pb analyses.

**Table 1:** List of Treatments to selected vegetables at Sargodha.

S. No	Symbols	Soil Amendment
1	T_0_	Control (clean Soil + Canal water irrigation)
2	T_1_	Cow manure at the rate of 20 tons ha^−1^ + canal waterirrigation
3	T_2_	Poultry manure at the rate of 3 tons ha^−1^ + canal water irrigation
4	T_3_	Leave compost at the rate of 50 tons ha^−1^ + canal water irrigation
5	T_4_	Sugarcane bagasse ash at the rate of 40 tons ha^−1^ + canal water irrigation
6	T_5_	Fertilizers (Nitrate at the rate of 100 kg ha^−1^) + canal water irrigation
7	T_6_	Clean soil + wastewater treatment
8	T_7_	Cow manure at the rate of 20 tons ha^−1^ + wastewater irrigation
9	T_8_	Poultry manure at the rate of 3 tons ha^−1^ + wastewater irrigation
10	T_9_	Leave litter at the rate of 50 tons ha^−1^ + waste water irrigation
11	T_10_	Sugarcane bagasse ash at the rate of 40 tons ha^−1^ + waste water irrigation
12	T_11_	Fertilizers (Nitrate at the rate of 100 kg ha^−1^) + waste water irrigation

#### Sowing

Following winter vegetables (*Daucus Carota, Brassica Oleracea, Pisum Sativum, Solanum tuberosum, Raphanus Sativus****,**** Spinacia Oleracea*) were grown in October 2018–2019 (Table 2). Seeds for each vegetable were sown in separate plots filled with compost. Vegetables were harvested after 60 days of germination.

**Table 2:** Selected vegetables are grown in Sargodha from waste water and canals water with fertilizers.

Scientific Name	English Name	Portion used for analysis
*Daucus carota*	Carrot	Root
*Brassica oleracea*	Cauliflower	Fruit
*Spinacia oleracea*	Spinach	Leaves
*Raphanus Sativus,*	Raddish	Root
*Solanum tuberosum*	Potato	Root
*Pisum sativum*	Pea	Fruit

The edible portions of the vegetables were taken for Pb analysis.

#### Irrigation

Vegetables were appropriately irrigated with canal water and wastewater at the intervals of 7–14 days for 2 yrs.

#### Sampling

##### Water, soil and vegetables sampling

Five replicates of wastewater and canal water samples that were used for irrigation purpose were collected and transferred in to the water analysis lab and stored at 4 °C before analysis. Before manure addition, five replicates of soil samples were taken at 0–30 cm depths for physical analysis. After adding manure and nitrate, soil samples with five replicates were collected for Pb and physical analysis at 0–30 cm depths by digging out a stainless steel auger. After that, the soil samples were dried in the air and stored in plastic bags for future study.

The mature edible portions of vegetables were collected randomly from the study site. Root vegetables consist of carrot (*Daucus carota),* radish (*Raphanus sativus*), potato (*Solanum tuberosum*). Leaf containing vegetables spinach (*Spinacia oleracea)* and cauliflower *(Brassica oleracea).* Seed containing vegetables *(Pisum sativum).* Vegetable samples were collected randomly, with five replicates from each plot irrigated with canal water and wastewater. Samples were washed through the water to remove soil particles, and vegetable samples were dry in air at 80 °C to constant mass. Dried samples were crushed using a mortar and pestle. After that, the samples were air-dried and kept in sealed paper bags with labels.

##### Pb analysis of water, soil, and vegetables samples

To investigate the level of Pb in water, 20 ml of water samples were digested with pure HNO_3_ (15 ml) until colorless at 80 °C. After filtration using whatman Filters #42 paper, distilled water was added to make the volume up to 50 ml. The dry powdered soil and food crop samples (1 g) were digested with 5 ml of concentrated HNO_3_ and aqua regia (15 ml, 70% Conc. HNO_3_ and 65% HClO_4_; 2:1) and heated until white fumes appeared at 80 °C. After filtering, the digested mixture was diluted with purified water to make a final amount of 50 ml.

The lead level in water, soil, and vegetable samples was analyzed using a calibrated atomic absorption spectrophotometer (Shimadzu Co., Ltd., Japan). The Samples were evaluated against the National Institute of Standard Technology’s Standard Reference Material (SRM) 1570 for lead to ensure precise and accurate results. The conditions for this process involved a wavelength of 283.3 nm, a slit width of 0.7 nm, a lamp current of ten mA, an airflow rate of 15 L/min, an acetylene flow rate of 2.0 L/min, and a flame height of 7 mm. Each sample was tested for Pb three times. All results were in line with worldwide standards.

#### Quality control

All the chemical reagents used for this study were sourced from Sigma Aldrich, Merck (Germany), and BDH (U.K.). These experiments used only Pyrex glassware. This glassware was meticulously cleaned with Max liquid detergent and then oven-dried at 100 °C for 1 h to ensure cleanliness.

#### Pollution load index

The pollution load index (PLI) is calculated using Eq. (1)^18^.

1 $$PLI = \left( M \right)IS/M)RS$$

M is the metal content (mg/kg), I.S. is the tested soil metal content, and RS. is the soil metal reference value. PLI 0 or < 1 implies no metal pollution, while PLI > 1 indicates high metal pollution and reduced soil value.

#### Bio-concentration factor

The bio-concentration factor (BCF) compares soil and an edible plant metal concentration is calculated through Eq. (2).

2 $$BCF = Metal \, in \, vegetables/Metals \, in \, soil$$

#### Soil enrichment factor (E.F.)

Soil enrichment factor (E.F) is utilized for estimating the accumulation of heavy metals concentration in soil as determined by the Eq. (3).

3 $$EF = \frac{{Metals \, \left( {Vegetables/Soil} \right) \, Sample}}{{Metals \, \left( {Vegetables/Soil} \right) \, Reference \, value}}$$

The reference values for lead (Pb) in soil (8.15 mg/kg) and vegetable (3 mg/kg) samples.

#### Daily intake of lead (DIM)

The daily intake of heavy metal (DIM) was calculated by applying this Eq. (4).

4 $$DIM = C\; \times \;Cf\; \times \;D/F$$

where C is the vegetable metal concentration; Cf is the conversion factor, 0.085. D is the daily food consumption, 0.345 kg/person/day and B is the normal adult weight, 65 kg.

#### Health risk index (HRI)

The health risk index for metal consumption from contaminated vegetables was calculating using the Eq. (5).

5 $$HRI = DIM/RfD$$

R_f_D for the lead was 0.004

### Statistical analysis

The result from each variable was statistically examined after the samples were analyzed with Microsoft Excel and Minitab 16. The means of soil, crop and water samples were compared using a three-factor factorial design (three-way ANOVA), and when the *p*-value was less than 0.05, it was concluded that the difference between the means had statistical significance. The mean of different treatments was compared through analysis of variance. Correlation analysis was used to find out Pb movement from soil to vegetables. The mean values for each treatment were compared using an analysis of variance.

### Statement regarding plant guidelines

Research regarding this manuscript was conducted inside the Department of Botany, University of Sargodha, Sargodha, Pakistan and during this research no animal or plant was harmed and no work was performed on new germline or new species. Furthermore, the plants were treated after the approval and guidelines from the departmental permission and standard methods were used to analyze the plant materials.

## Results

### Concentration of lead (Pb) in the irrigated water

Lead values in water showed significant (*P* < 0.05) variations in yr, water type, and water type × yr (Table 3). The Pb mean for Crop lead (Pb) versus treatment, vegetables and yr (Table 4). The concentration of lead contents ranged from 0.0329 to 0.0473 mg/L in freshwater and 0.1500–0.2847 mg/L in wastewater during both yrs (Table 5).

**Table 3:** Analysis of variance of water lead (Pb) to selected vegetables.

Source	DF	Mean Square
Water type	1	0.163972***
Season	1	0.011159**
Water type * Season	1	0.007700**
Error	16	0.000181
Total	19	

*, **, and ***indicates significant at 0.05, 0.01, and 0.001 levels.

**Table 4:** Analysis of variance of Crop lead (Pb) versus treatments, vegetables, and years.

Source	DF	Mean Square
Treatments	11	103.479*****
Vegetables	5	114.985*****
Years	1	45.591****
Treatments * vegetables	55	2.788***
Treatments * years	11	0.838***
vegetables * years	5	18.956****
Treatments * vegetables * years	55	1.609***
Error	576	0.005
Total	719	

*, ** and ***indicates significant at 0.05, 0.01, and 0.001 levels.

**Table 5:** Mean concentrations of lead (Pb) (mg/L) in diverse source of water during both years.

Variable	Years	Water type	Mean	SE Mean	Minimum	Maximum
Lead (Pb)	1st	FW	0.0361	0.0011	0.0329	0.0393
	1st	WW	0.1780	0.0086	0.1500	0.2000
Lead (Pb)	2nd	FW	0.0441	0.0011	0.0409	0.0473
	2nd	WW	0.0264	0.0082	0.2386	0.2847

### Crop lead (Pb) versus treatments, vegetables, and years

#### Concentration of lead (Pb) in soil irrigated with wastewater

**Figure 1:**
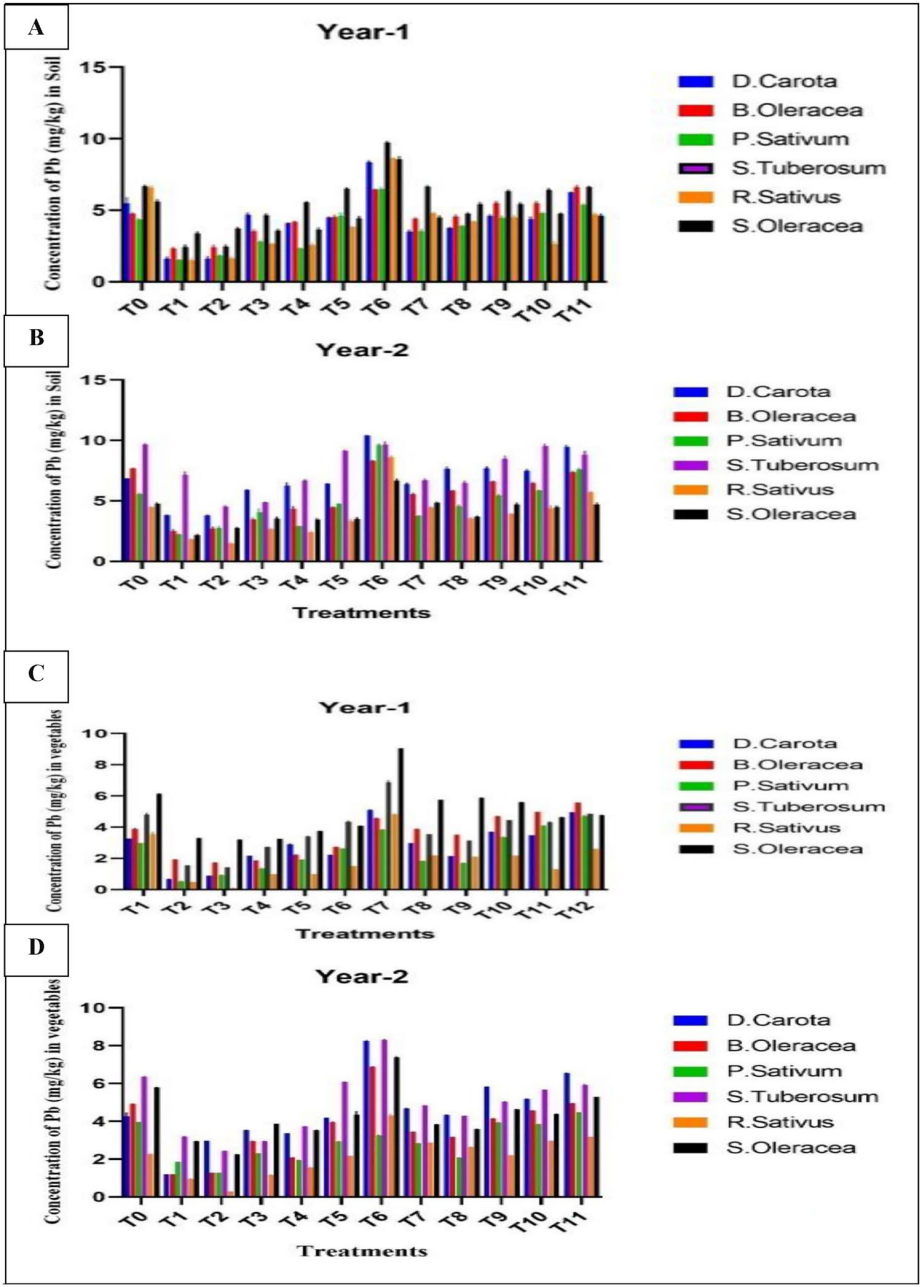
(**A**–**D**) Difference in the value of Lead (Pb) (mg/kg) in soils, Concentration of lead in soil for year-1 (**A**), Concentration of lead in soil for year-2 (**B**), Concentration of lead in vegetables for year-1 (**C**), Concentration of lead in vegetables for year-2 (**D**).

The data for analysis of variance for soil lead (Pb) value showed highly significant variation (*P* < 0.05) among treatments, vegetables, yrs, and their interactions like treatments × vegetables, treatment × yrs, vegetables × yrs, treatments × vegetables × yrs (Table 6). Soil lead (Pb) content varied for various treatments from 1.476 to 10.407 mg/kg during both yrs (Fig. 1A). The higher lead (Pb) values (10.48 mg/kg) were analyzed in *Daucus carota* during 2nd yr and treatment T_6_ (Clean soil + wastewater treatment). In comparison, the lower lead (Pb) values (1.48 mg/kg) were analyzed in *Raphanus sativus* during 2nd yr in treatment T_3_ (Leave compost at the rate of 50 t ha^−1^ + canal water irrigation) (Fig. 1B). The results show the mean concentration of lead (Pb) in vegetables and soil during both yrs for all treatments (Table 7). Mean value of lead (Pb) for all treatments in soil was 4.560 mg/kg and 5.476 mg/kg for 1st and 2nd yrs (Table 8).

**Table 6:** Analysis of variance of soil lead (Pb) versus treatments, vegetables, and years.

Source	DF	Mean Square
Treatments	11	159.003****
Vegetables	5	120.869***
Years	1	150.931****
Treatments * vegetables	55	3.718**
Treatments * years	11	2.148**
vegetables * years	5	41.578***
Treatments * vegetables * years	55	2.385**
Error	576	0.072
Total	719	

*, **, and ***indicates significant at 0.05, 0.01, and 0.001 levels.

**Table 7:** Mean concentrations of lead (Pb) (mg/kg) in vegetables and soil during both years for all treatments.

Variable	Treatments	Mean	SE Mean	Minimum	Maximum
Crop Pb	T0	4.349	0.161	2.277	6.459
	T1	1.651	0.127	0.473	3.307
	T2	1.564	0.123	0.087	3.211
	T3	2.428	0.118	0.977	3.872
	T4	2.619	0.117	0.983	3.760
	T5	3.434	0.160	1.490	6.093
	T6	6.056	0.246	3.186	9.071
	T7	3.559	0.141	1.820	5.770
	T8	3.214	0.150	1.687	5.889
	T9	4.146	0.146	2.156	5.853
	T10	4.121	0.144	1.289	5.675
	T11	4.817	0.135	2.587	6.568
Soil Pb	T0	6.071	0.194	4.174	9.874
	T1	2.701	0.198	1.038	7.817
	T2	2.637	0.124	1.226	4.882
	T3	3.865	0.130	2.453	5.980
	T4	4.031	0.187	2.135	6.916
	T5	4.996	0.206	3.041	9.306
	T6	8.459	0.169	6.037	10.478
	T7	4.920	0.147	3.046	6.941
	T8	4.857	0.160	3.166	7.977
	T9	5.639	0.178	3.796	8.913
	T10	5.554	0.225	2.190	9.988
	T11	6.484	0.203	4.238	9.856

**Table 8:** Mean Concentrations of lead (Pb) (mg/kg) in vegetables and soil for both years.

Variable	Years	Mean	SE Mean	Minimum	Maximum
Soil lead (Pb)	1	4.5600	0.0938	1.0380	9.9800
	2	5.476	0.119	1.275	10.478
Crop lead (Pb)	1	3.2448	0.0890	0.0934	9.0639
	2	3.7481	0.0896	0.2654	8.3870

#### Concentration of lead (Pb) in vegetables irrigated with wastewater

The data for analysis of variance for vegetable lead (Pb) value showed significant variation (*P* < 0.05) among treatments, vegetables, yr, and their interactions like treatments × vegetables, treatments × yrs, vegetables × yrs, treatments × vegetables × yrs (Table 6). Vegetables lead (Pb) content varied for various treatments from 0.097 to 9.40 mg/kg during both years in soil (Fig. 1A,B). The higher lead (Pb) values (9.40 mg/kg) were analyzed in *Spinacia oleracea* during 2nd yr and treatment T_5_ (Nitrate at 100 kg ha^−1^ + canal water irrigation). The lower lead (Pb) values (0.097 mg/kg) were analyzed in *Raphanus sativus* during 2nd yr and in treatment T_2_ (Poultry manure at the rate of 3 t ha^−1^ + canal water irrigation) (Fig. 1C,D). Mean value of lead (Pb) for all treatments in vegetables was 3.244 mg/kg and 3.74 mg/kg for 1st and 2nd yrs (Table 8).

### The pollution load index (PLI) of lead (Pb) in respective samples of vegetables

**Figure 2:**
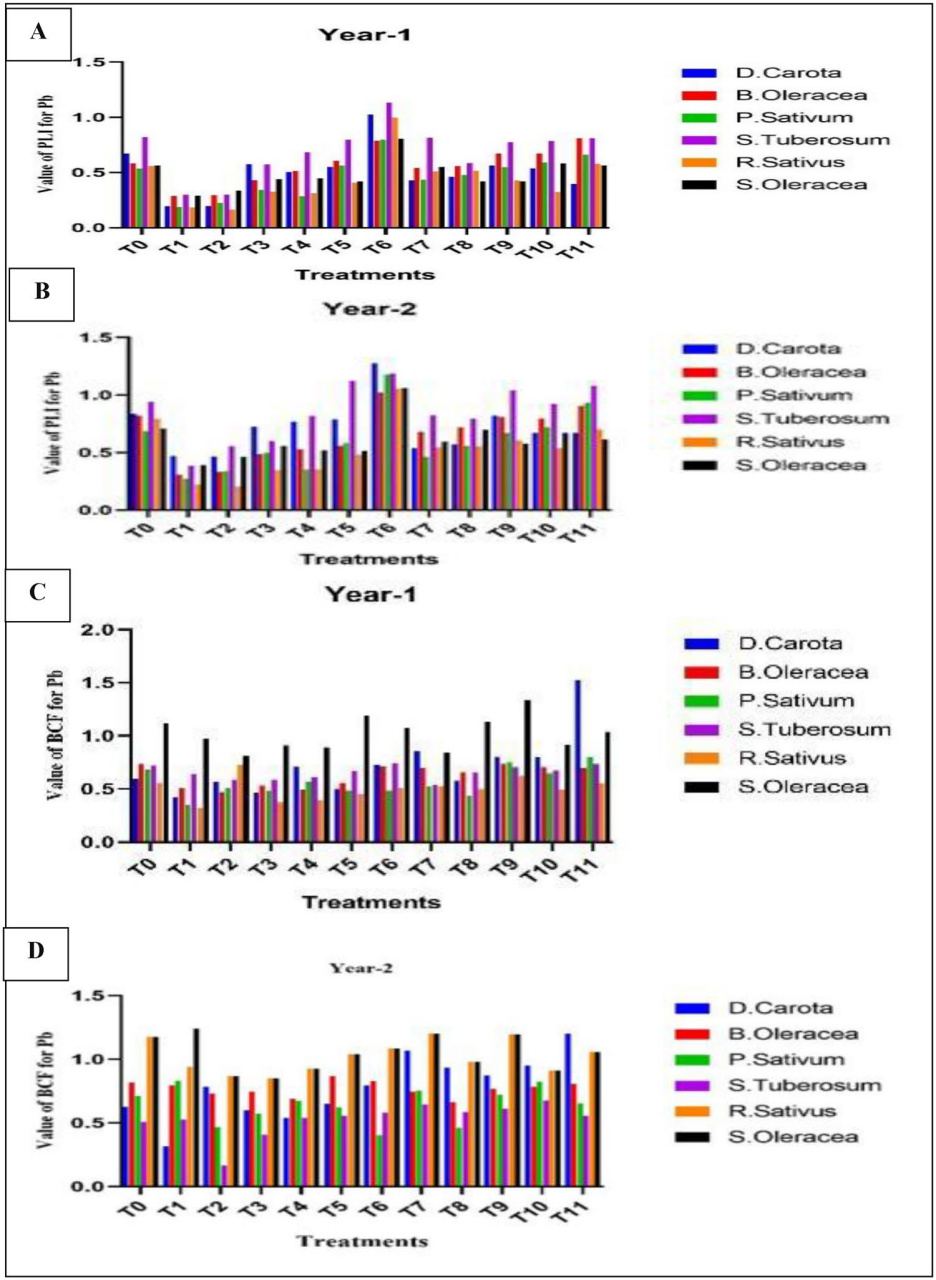
(**A**–**D**) Difference in the value of Lead (Pb) (mg/kg), Difference in the value of PLI in year-1 (**A**), Difference in the value of PLI in Year-2 (**B**), Difference in the value of BCF in year-1 (**C**), Difference in the value of BCF in year-2 (**D**).

The lead PLI value ranged from 0.0729 to 1.525 depending on the vegetables, yr, and treatment. A lower level of PLI (0.0723) was observed in *Raphanus sativus* during 1st yr in treatment T_2_. In contrast, a higher PLI (1.525) value was observed in *Daucus Carota* during 1st yr of treatment T_11_ (Fig. 2A,B). According to treatments, higher mean value of PLI for lead (Pb) in treatment T_6_ during 2nd yr was 1.08 (Table 9) while according to vegetables, the mean value of lead PLI was higher in *S. tuberosum* was 0.938 (Table 10).

**Table 9:** Mean for analysis of Value of PLI on the base of treatments in both years.

Years	Treatments											
	T_0_	T_1_	T_2_	T_3_	T_4_	T_5_	T_6_	T_7_	T_8_	T_9_	T_10_	T_11_
1st	0.683	0.260	0.278	0.447	0.457	0.580	0.986	0.559	0.543	0.629	0.581	0.698
2nd	0.797	0.402	0.368	0.501	0.531	0.646	1.08	0.648	0.647	0.754	0.781	0.893

**Table 10:** Mean Value of PLI in vegetables for all treatments during both years.

Years	Vegetables					
	*D.carota*	*B.Oleracea*	*P.Sativum*	*S.Tuberosum*	*R.Sativus*	*S.Olerace*
1st	0.540	0.558	0.470	0.703	0.492	0.588
2nd	0.839	0.668	0.604	0.938	0.477	0.503

### Bio-concentration factor of lead (Pb) in respective samples of vegetables

The value of the lead bio-concentration factor varied from 0.057 to 1.28, and this variation depended on the vegetables, yr, and treatment**.** *Spinacia oleracea* had a higher value of BCF was 1.28 at treatment T_8_ in 1st yr. In comparison, *Raphanus sativus* had a lower BCF value at treatment T_3_ (Leave compost at the rate of 50 t ha^−1^ + canal water irrigation) in 1st yr (Fig. 2C). Higher mean value of BCF for lead in treatment recorded at T_1_ in *S. oleracea* 2nd yr (Fig. 2D). Higher mean value of BCF for lead in treatment T_11_ (Nitrate at the rate of 100 kg ha^−1^ + wastewater irrigation) and 1st yr was 0.889 (Table 11). The average value of lead BCF was higher in *S. Oleracea* was 1.059 (Table 12).

**Table 11:** Mean Value of BCF on the base of treatments in both years.

Years	Treatments											
	T_0_	T_1_	T_2_	T_3_	T_4_	T_5_	T_6_	T_7_	T_8_	T_9_	T_10_	T_11_
1st	0.748	0.536	0.500	0.55 7	0.610	0.641	0.707	0.66 9	0.543	0.660	0.831	0.717
2nd	0.842	0.793	0.637	0.711	0.731	0.829	0.800	0.867	0.765	0.858	0.854	0.899

**Table 12:** Mean Value of BCF in vegetables for all treatments during both years.

Years	Vegetables					
	*D.carota*	*B.Oleracea*	*P.Sativum*	*S.Tuberosum*	*R.Sativus*	*S.Olerace*
1st	0.710	0.644	0.560	0.654	0.446	1.019
2nd	0.777	0.749	0.640	0.528	1.044	1.059

### The enrichment factor (EF) of lead (Pb) in respective samples of vegetables

**Figure 3:**
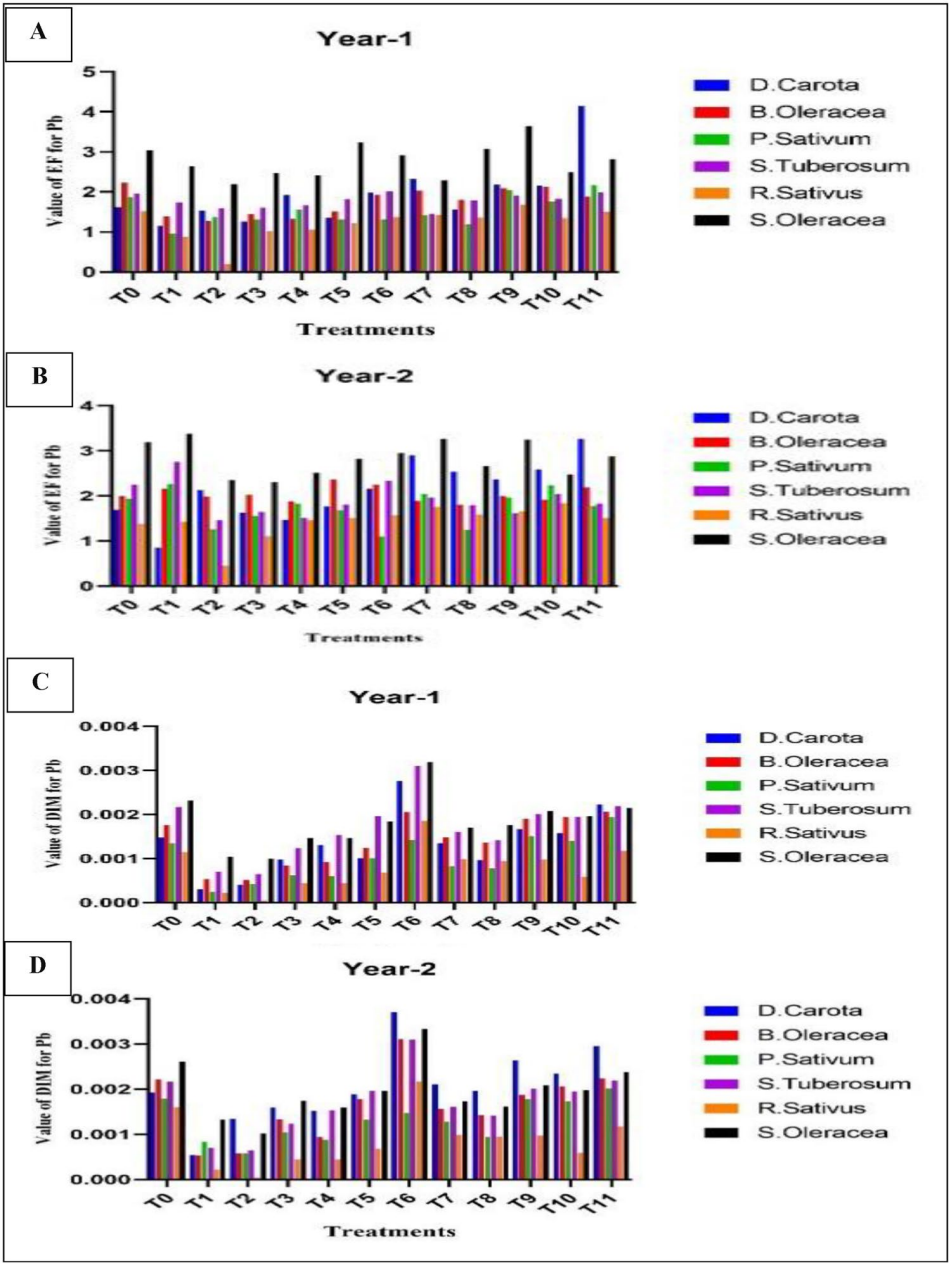
(**A**–**D**) Difference in EF and DIM, Difference in the value of EF in year-1 (**A**), Difference in the value of EF in year-2 (**B**), Difference in the value of DIM in year-1 (**C**), Difference in the value of DIM in year-2 (**D**).

The enrichment factor (EF) value for lead varied from 0.156 to 3.49. In the 1st yr in treatment T_8_, *Spinacia oleracea* had maximum EF value (3.49) (Fig. 3A). In contrast, *Raphanus sativus* had the minimum EF value (0.156) during 1st yr in treatment T_3_ (Leave compost at the rate of 50 t ha^−1^ + canal water irrigation) (Fig. 3B). The highest mean EF value for lead was noted (4.144), for the 1st yr in treatment T_11_ (Nitrate at the rate of 100 kg ha^−1^ + wastewater irrigation) (Table 13). In the case of *S. oleracea*, the average value of lead EF (3.19) was higher (Table 14).

**Table 13:** Mean Value of EF on the base of treatments in both years.

Years	Treatments											
	T_0_	T_1_	T_2_	T_3_	T_4_	T_5_	T_6_	T_7_	T_8_	T_9_	T_10_	T_11_
1st	1.161	1.150	1.152	1.255	1.917	1.354	1.983	2.319	1.55	1.186	2.161	4.414
2nd	1.695	0.853	2.128	1.625	1.466	1.766	2.155	2.903	2.538	2.370	2.581	3.260

**Table 14:** Mean Value of EF in vegetables for all treatments during both years.

Years	Vegetables					
	*D.carota*	*B.Oleracea*	*P.Sativum*	*S.Tuberosum*	*R.Sativus*	*S.Olerace*
1st	1.615	2.224	1.856	1.951	1.513	3.037
2nd	1.695	1.994	1.931	2.252	1.377	3.197

### Daily intake (DIM) of lead (Pb) in respective samples of vegetables

Lead DIM was measured from 0.00004 to 0.0040 mg/kg/day. The lowest value for daily intake of Pb, 0.00004 mg/kg/day, was found in *Raphanus sativus* during 1st yr in treatment T_3_ (Leave compost at the rate of 50 t ha^−1^ + canal water irrigation), but the highest value for daily intake of Pb 0.0040 mg/kg/day was found in *Spinacia oleracea* during 1st yr in treatment T_6_ (Cow manure at the rate of 20 t ha^−1^ + wastewater irrigation) (Fig. 3C). The highest value for daily intake of Pb was found in *D. carota* during 2nd yr in treatment T_5_ (Fig. 3D). The higher mean value of DIM for lead in treatment T_9_ during 1st yr was 1.186 mg/kg/day (Table 15). The mean value of daily intake for lead was higher in *S. Oleracea* was 0.0030 mg/kg/day (Table 16).

**Table 15:** Mean Value of DIM on the base of treatments in both years.

Years	Treatments											
	T_0_	T_1_	T_2_	T_3_	T_4_	T_5_	T_6_	T_7_	T_8_	T_9_	T_10_	T_11_
1st	0.0014	0.0003	0.0004	0.0009	0.0013	0.0010	0.0027	0.0013	0.0009	1.186	0.0016	0.0015
2nd	0.0019	0.0054	0.0013	0.0015	0.0015	0.0018	0.0021	0.0021	0.0019	0.0026	0.0023	0.0029

**Table 16:** Mean Value of DIM in vegetables for all treatments during both years.

Years	Vegetables					
	*D.carota*	*B.Oleracea*	*P.Sativum*	*S.Tuberosum*	*R.Sativus*	*S.Olerace*
1st	0.0014	0.0017	0.0013	0.0021	0.0011	0.0023
2nd	0.0019	0.0022	0.0017	0.0028	0.0014	0.0030

### Health risk index (HRI) of lead (Pb) in respective samples of vegetables

**Figure 4:**
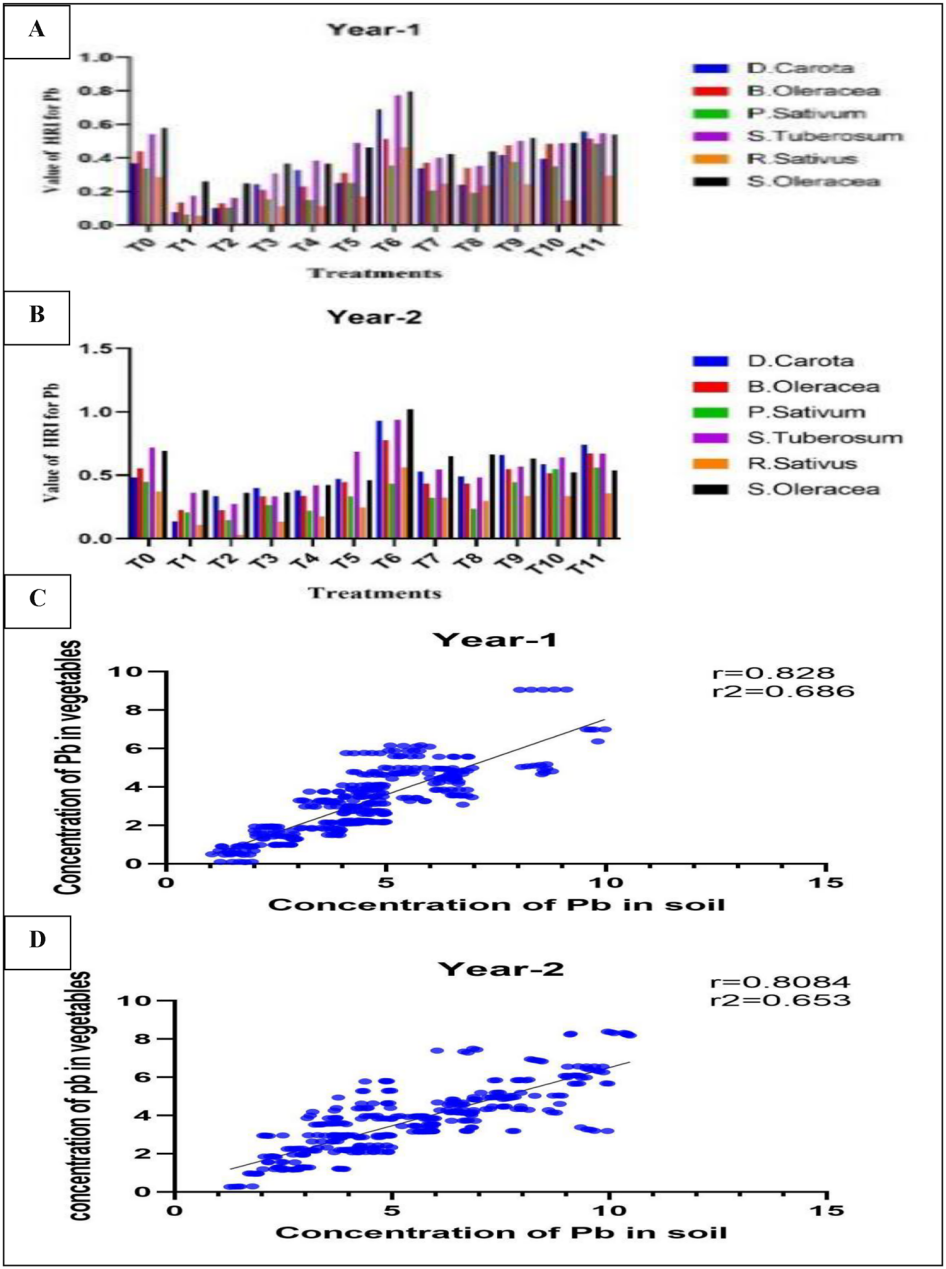
(**A**–**D**) Correlation and value difference between concentration of lead in vegetables and soil for all treatments during both years. Difference in the value of HRI in year-1 (**A**), Difference in the value of HRI in year-2 (**B**), Correlation between vegetables and soil (**C**), Correlation between vegetables and soil in year-2 (**D**).

Lead HRI was measured in the range of 0.0105–1.022. The lowest HRI value (0.0105) was found in *Raphanus sativus* 1st yr treatment T_3_ (Leave compost at the rate of 50 t ha^−1^ + canal water irrigation), but the higher HRI (1.022) was found in *Spinacia oleracea* in 1st yr treatment T_7_ (Cow manure at the rate of 20 t ha^−1^ + wastewater irrigation) (Fig. 4A,B). Higher mean value of health risk index for lead in treatment T_6_ and 2nd yr was 0.733 (Table 17). Mean value for health risk index of lead was greatest in *S. Oleracea* was 0.579 (Table 18).

**Table 17:** Mean Value of HRI in vegetables on the base of treatments in both years.

Years	Treatments											
	T_0_	T_1_	T_2_	T_3_	T_4_	T_5_	T_6_	T_7_	T_8_	T_9_	T_10_	T_11_
1st	0.4437	0.1469	0.1444	0.2321	0.2708	0.3220	0.6365	0.3683	0.3379	0.4410	0.3971	0.4893
2nd	0.5183	0.2280	0.2102	0.3155	0.3219	0.4448	0.7332	0.4307	0.3906	0.4981	0.5191	0.5990

**Table 18:** Mean Value of HRI in vegetables for all treatments during both years.

Years	Vegetables					
	*D.carota*	*B.Oleracea*	*P.Sativum*	*S.Tuberosum*	*R.Sativus*	*S.Olerace*
1st	0.3676	0.4385	0.3359	0.5417	0.2869	0.5790
2nd	0.5112	0.4581	0.3462	0.5526	0.2493	0.4873

### Scatter plot analysis for lead (Pb) concentration in soils and vegetables irrigation with waste

Scatter plot analysis was conducted to examine the lead concentration in soils and vegetables for each treatment. A positive correlation was identified between concentration of lead (Pb) in the soil and in the vegetables. A notably significant positive correlation was observed between soils and vegetables. However, the regression analysis also indicated a non-significant (*P* > 0.05) value for the lead (Pb) in the soil, and similarly, a non-significant (*P* > 0.05) regression in the vegetable samples (Fig. 4C,D).

## Discussion

### Lead (Pb) levels in wastewater

In this current analysis, the concentrations of lead in wastewater and canal water were higher, ranging from 0.044 to 00.21 mg/L, compared to the previously reported values^18^. observed that lead contents (7.15 mg/L) in wastewater were significantly higher than the current analysis finding.

Lead is primarily consumed by plants through the topmost layer of soil. The presence of lead (Pb) in the water sources used for crop irrigation may have resulted in the decreased availability of lead (Pb) for plant absorption^19^. External factors such as pollution from heavy traffic or the presence of lead (Pb) in crop irrigation water sources for crops may have impacted the availability of lead (Pb) to plants. Lead accessibility for plant uptake may also be decreased by environmental contamination brought on by vehicle emissions.

### Lead (Pb) levels in soil after treatment with various fertilizers and wastewater

In one of study observed that lead contents in soil range from 1.95 to 4.9 mg/kg, which had similar mean values of lead (Pb) content in treatments T_2_, T_3_, T_4_, T_5_, T_7_, T_8_, T_10_ (1st yr) and treatments T_1_, T_2,_ T_3_, T_4_, (2nd yr) while more significant in all other treatments^20^.

Irrigating soil with canal water and wastewater significantly increases the concentration of lead (Pb). This revealed that the consistent use of wastewater cause to the accumulation of lead (Pb) in the soil. Results found that wastewater-irrigated soil increased the accumulation of lead contents in the soil’s upper layer. In a study it was described soil treated with cattle manure showed a lead (Pb) content between 3.6 and 5.9 mg/kg, similar to recent research findings^21^. These results can be affected by various factors, including soil type, crop growing techniques, and manure quality.

### Lead (Pb) levels in vegetables after treatment with various fertilizers and wastewater

Applying municipal solid waste (MSW), treatments increased the soil’s lead (Pb) levels by 97.2%. Applying 200 t of manure per hectare, Pb in the soil increased by roughly 14–278% compared to soil where no manure was used^22^. Mixing plant waste with manure reduced the effectiveness of removing Pb from polluted soils, suggesting it’s a beneficial way to absorb these elements and improve soil fertility. Treatments of decomposed poultry manure with pine waste decreased the remaining lead (Pb) percentage in soil from 294 mg kg^−1^ (1:0) to 276 mg kg^−1^ when applied at 10 t per hectare and 20 t per hectare^23^. It was observed that the lead (Pb) content in wastewater-irrigated vegetables increased by 28 mg/kg, showing a higher lead (Pb) content in all treatments and yrs in our current data^9^.

Using wastewater significantly increased the lead (Pb) levels in the soil that produced all these vegetables (*p* 0.05). Lead (Pb) contents in soil and vegetables increased after Pb-containing wastewater was applied^9^. It was reported that the average amount of lead (Pb) in the edible parts of vegetables ranged between 1.8 and 11 mg/kg, demonstrating higher lead build-up than our current data^24^. Lead (Pb) contents in spinach range from 1.0 mg/kg, which were lower in all treatments of spinach as compared to present finding. The lead (Pb) levels in vegetables irrigated with wastewater ranged from 32.2 to 34.2 mg/kg, which exceeds our present data for these vegetables^25^.

Lead contamination of the environment is being researched because lead entry into the food chain may affect human health and vegetable metabolism. The growing need for agricultural land increases nutrient movement, decreasing the soil’s essential nutrients. Using organic materials is vital for the long-lasting health of intensive systems. Many studies have shown the positive effects of applying organic matter to soil properties. Heavy use of cow manure significantly impacts the accumulation of Pb in the soil. The notable increase emphasizes the role of cow manure in large-scale farming. The lead (Pb) content decreases with the application of cow manure average lead (Pb) content in various food crops fertilized with poultry manure (varying from 0.41 to 11.4 mg per kg) was significantly higher than the current results^26^. In soil treated with poultry manure, the available lead content for plants was 10%, compared to 14% lead value in the untreated soil^27^.

Pb spread and transfer to the environment can be reduced by compost as compared to fresh manure. Applying compost manure in polluted soils has been shown to lower heavy Pb contamination and has proven beneficial and effective. According to WHO, the maximum safe level of lead (Pb) for human use is 0.3 mg per kg of dry weight^28^. Lead (Pb) levels in the edible parts of plants varied between 0.09346 and 9.0639. The lead content in these plant parts was above the safe limit. Lead levels decreased when manure was added, while they increased when wastewater was used. Ecological exposure, compost, and wastewater treatment are farming fields’ primary lead sources (Pb). Large clusters of industrial facilities, vehicle emissions, re-suspended roadway sand, and petrol engine systems all play a part in lead absorption.

A higher pollution load index (0.62 mg/kg) was reported compared to present study^29^. In most studies, lead (Pb), a major pollutant of concern, often exceeded a value of 1, The Pollution Load Index (PLI) increase can be attributed to various human activities. It was reported that the Pollution Load Index (PLI) for lead (Pb) was within the range of 0.49–0.61, similar results observed in treatments T_3_ and T_4_ in the 1st yr and T_1_ in the 2nd yr of our current study^30^. The PLI value experienced a decrease when a combination of fertilizers, namely poultry manure, cow manure, and NPK, was applied. The PLI value decreased and was observed to be less than 1 in all treatments throughout both years, except treatment T_6_ in the 2nd yr, which involved wastewater irrigation.

The bio-concentration factor of lead irrigated with canal water and wastewater, (Pb) in vegetables ranged from 0.18 to 0.20 and 0.18 to 0.19. These values indicated a lower average compared to the findings of our current study. The Bio-concentration Factor (BCF) in vegetables irrigated with wastewater was higher, with observed values ranging from 0.096 and 0.211 mg/kg^31^. Upon treating the soil with composted cattle manure at a rate of 10 t per hectare, the Bio-concentration Factor (BCF) value of lead (Pb) in plants was reduced to 7.6 mg/kg, ultimately similar to the control treatment (10.8 mg kg^1^)^23^. The application of manure reduced the total Pb content in the soil. Manure could reduce lead (Pb) availability in soil through complex structures that form between organic matter and Pb.

Alterations with high-pH organic substances may further decrease the absorption of lead (Pb)^23^. It was observed the enrichment factor (EF) value for lead (Pb) was 0.53, which is lower compared to our current study. The enrichment factor value in vegetables was 2.187, which is higher, and was reported for the accumulation in the edible parts of vegetables grown in soil irrigated with wastewater and fertilized with inorganic fertilizers^32^. Plants that transfer lead from the soil to their edible parts have an enrichment factor (EF) greater than 1. These consumable plant portions accumulate more lead (Pb) than the soil. Metals with a high enrichment factor (EF) value can quickly move into the consumable parts of plants^33^.

The daily Pb intake was calculated as 0.345 kg of vegetable for a 65 kg standard body mass. The daily average values for metal consumption was identified by^34^. The USEPA published suggested consumption for reference value for many metals^35^. Soil irrigated with wastewater had higher value of daily intake of Pb as compared to current research. Amount of daily intake of lead was reported 8.15 to 75.06 mg/day^36^ and similar results were analyzed by^37^. Results reported that lower value of daily intake of Pb for wastewater irrigated soil as compared to current research.

Value of daily intake of Pb recorded higher (3.51 mg/day/kg) in body weight as compared to the present data^31^. Humans, especially children, can develop mental and neurological problems as a result of lead poisoning. Higher value of the health risk index than our current research was reported^38^. The health risk index (HRI) levels in canal water and wastewater-irrigated soil ranged from 8.83 to 9.09 mg/kg, exceeding the acceptable limits^39^.

## Conclusions

In response to dwindling fresh water resources, farmers have increasingly relied on wastewater for enhancing agricultural productivity. Wastewater also contains vital nutrients for plant growth, in addition to harmful chemicals. The results indicate that soils were enriched with lead (Pb). Applying organic fertilizers reduced the lead (Pb) concentration in the soil, which was generated by the usage of wastewater and its transfer to edible crops. This study examined the possible health effects resulting from lead (Pb) pollution of soil and vegetables. The addition of wastewater resulted in a health risk index (HRI) of less than 1, meaning that lead (Pb) pollution has no negative consequences. Treatment T_6_ (wastewater + Soil without manure) contains the highest amount of chromium in soil among all treatments. However, analyses have shown that both soil and crops contain lead concentrations in the safe levels recommended by the Food and Agriculture Organization (FAO) and the World Health Organization (WHO). In order to prevent lead poisoning in soils and crops and potential health risks in the vicinity of the study zone, it is imperative to regularly monitor water pollution and treatment. Addition of mineral and organic fertilizers can reduce the Pb accumulation in vegetables.

### Supplementary Information

Supplementary Information.

## Data Availability

Data is provided within the manuscript or supplementary information files.
